# Change in functional disability and its trends among older adults in Korea over 2008–2020: a 4-year follow-up cohort study

**DOI:** 10.1186/s12877-023-03867-5

**Published:** 2023-04-06

**Authors:** Van Cuong Nguyen, Gwi-Ryung Son Hong

**Affiliations:** School of Nursing, Hanyang University, Seoul, Korea

**Keywords:** Demographics, functional disability, Logistic regression, Longitudinal study, Prevalence

## Abstract

**Background:**

The prevalence of functional disabilities, including difficulties in performing activities of daily living (ADLs) and instrumental activities of daily living (IADLs), increased significantly in recent years and burdened the healthcare system.

**Methods:**

We analysed data from Korean Longitudinal Study of Aging (KLOSA) surveys, including participants aged 65 or older at baseline (2008), and participated in all 4-year follow-up periods in 2012, 2016, and 2020. A 4-year follow-up cohort study was applied to specify the change in functional disability and its trend over time among older adults. The generalized estimation equation (GEE) model was used to verify the uptrend of functional disability. Logistic regression analyses were applied to examine the influence of demographic and health parameters on the change in functional disability.

**Results:**

The prevalence of ADL disability was 2.24% at baseline, increased to 3.10% after four years, 6.42% after eight years, and reached 11.12% after 12 years, five times higher than that at baseline. For IADL disability, they were 10.67%, 10.61%, 18.18%, and 25.57%, respectively. The uptrend of ADL disability in persons aged 65–74 (1.77% at baseline, increased to 7.65% in 2020, 12-year change of 5.88%) was slower than in those aged 75 or older (4.22% at baseline, increased to 25.90% in 2020, 12-year change of 21.68%). IADL disability were consistent with this. The high ADL/IADL disability rate was also present among persons with poor health status, physical inactivity, depression, dementia, and multiple chronic diseases. The relative risk of ADL/IADL disability in persons with a history of functional disability was significantly higher than in those without historical disabilities.

**Conclusion:**

The study verified the change in functional disability and its upward trend over time by older adults’ demographic and health parameters. Functional disability was relatively flat tending to increase slowly during the early years but increased rapidly in the following years. Factors that strongly influenced the change in prevalence and the uptrend of functional disability were advanced age, living alone, being underweight or obese, poor health status, physical inactivity, depression, dementia, having multiple chronic diseases, and especially having a historical disability.

## Background

Population aging in Korea has taken place rapidly in recent years. According to 2021 data from the South Korea Age structure [1], the number of persons aged 65 or older accounted for 15.66%. This rate was higher than the world average (9.69% [2]). The Korea Herald has predicted that the Korean population aged 65 and older may reach 43.9% by 2050 [3]. The rapid growth of the population aged 65 or older has led to undesirable outcomes such as increased dependency, caregiver burden, and public healthcare costs [4, 5]. This has placed considerable pressure on policymakers related to healthcare for older adults.

Along with the global population aging, the prevalence of functional disabilities, including difficulties in performing activities of daily living (ADLs) and instrumental activities of daily living (IADLs), has increased significantly in recent years and affected the quality of life [5]. Deficiencies in performing ADLs, which included basic activities, represent a more severe stage of disability. In contrast, deficiencies in performing IADLs, which included more complex activities, identify disabilities at an earlier stage. Therefore, the prevalence of IADL disability was often higher than that of ADL disability [6]. A previous study found that the proportion of older population of south-eastern Poland with at least one ADL limitation was 17% and 36% with at least one IADL limitation [7]. In China, these rates were 10% and 26%, respectively [8]. Functional decline increased with age [8–12] and changed between gender [5]. The rates of ADL and IADL disabilities among older males in Germany (14.1% and 15.7%, respectively) were much lower than in females (20.0% and 28.2%, respectively) [5]. Similarly, these rates in Italy were 13.6% and 17.2% for older males; 25.3% and 35.7% for older females, respectively [5]. Recent studies have also shown that functional decline in older adults strongly depends on educational status, physical activity, cognitive function, and chronic disease status [13–18]. However, the historical disability has not been fully and systematically assessed by various factors such as gender, educational status, physical activity, and cognitive functions. Functional decline has severely strained the national healthcare system and increased medical costs [19, 20].

Identifying the trends of disability was essential and has received much attention recently [4–6, 21–24]. Previous studies have shown that a decreasing trend of functional disability occurred in most countries in recent decades. The prevalence of functional disability was stable in the United States during the period 1998–2008 [21], while it decreased in European countries for the period 2004–2013 [5] and in China for the period 1997–2006 [6]. However, these studies were based on cross-sectional data, so no trends in functional disability in older adults could be observed over time. Longitudinal studies to identify disability trends among older adults have been relatively scarce, especially for older Korean adults. Although several longitudinal studies were performed [6, 8, 25], due to the short continuous follow-up, these studies have only shown a tendency toward disability in the early stages, not observed changes in disability among older adults as they entered later life. Furthermore, factors influencing the change in functional disability, such as chronic disease status, cognitive decline, and historical disability, have not been comprehensively evaluated in these studies.

In this paper, we performed a 4-year follow-up study to determine the prevalence of functional disability and its trend over time among older adults using a nationally available dataset. The factors affecting the change and trend of functional disability were also simultaneously examined.

## Methods

### Study design and participants

The Korean Longitudinal Study of Aging (KLOSA) data was an extensive dataset collected based on a representative nationwide survey every two years through individual interviews [26, 27]. The survey targeted community-dwelling adults aged 45 or over and covered eight topics affecting adults’ economic and social activities: demographics, family, health, employment, income, assets, subjective expectations, and life satisfaction. The first wave was surveyed in 2006 with 10,254 participants but the health parameters of the participants were not fully covered such as excluding the CESD-10 scale to define depression. Moreover, many participants have not completed the health survey (5821 persons). The second wave was conducted in 2008 and included participants with complete health parameters. A total of eight surveys have been conducted up to 2020. Detailed information on these surveys is available on the survey organization’s website [26].

**Fig. 1:**
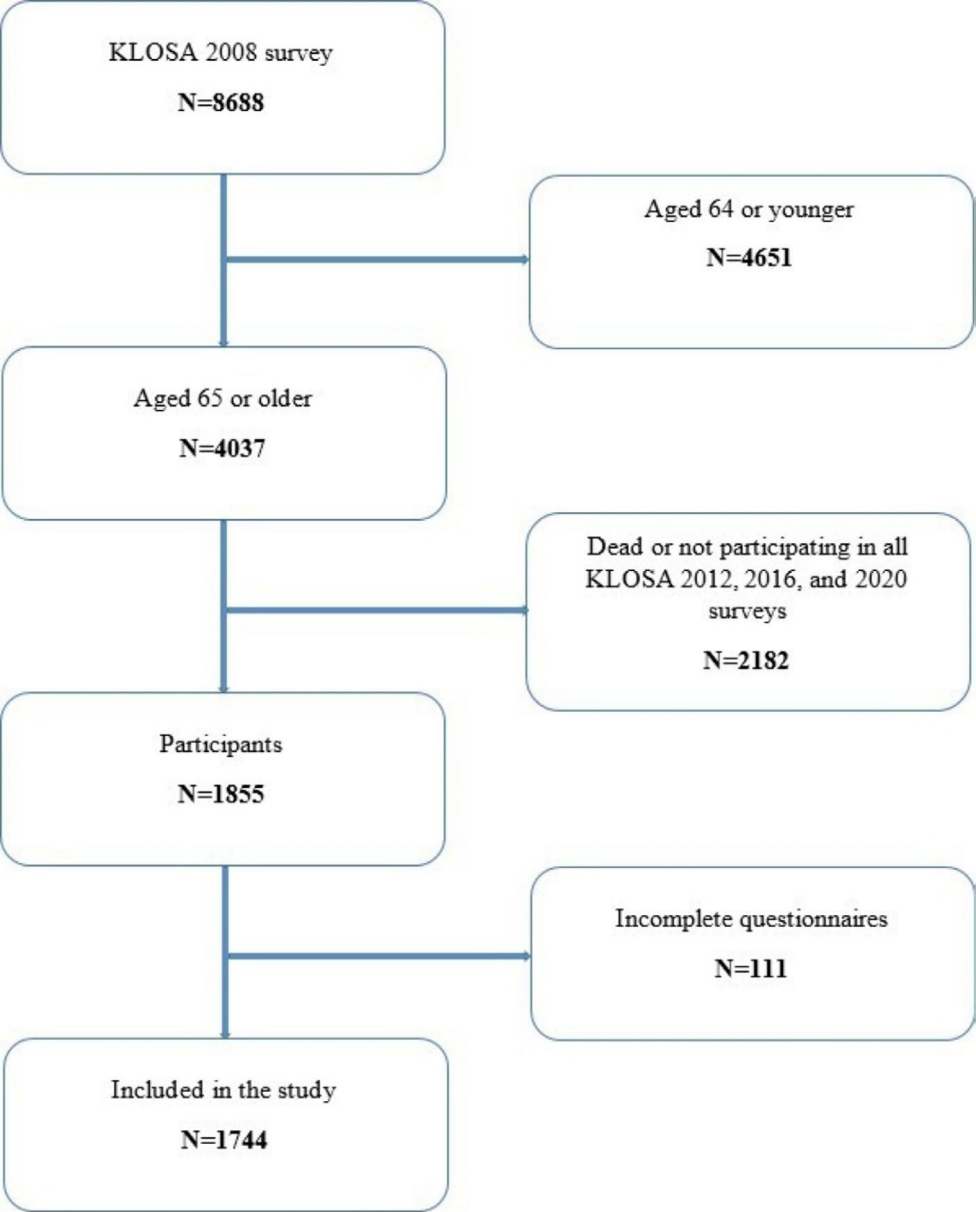
Flow diagram of the study population *KLOSA* Korean Longitudinal Study of Aging; *N* Number of participants

For research purposes, we conducted a 4-year follow-up cohort study with a 2008 baseline and three 4-year follow-up periods in 2012, 2016, and 2020. To account for participants, we included all persons aged 65 or older (older adults) who participated in and completed questionnaires in all four KLOSA surveys. The final sample was composed of 1744 older participants. Figure 1 shows the participant selection and drop-out process.

### Functional disability

KLOSA surveys considered the ADL scale from seven indicators: dressing, washing, bathing, eating, getting in/out of bed, toileting, and managing bladder/bowel [18, 26]. The IADL scale included ten instrumental activities: grooming, household chores, preparing meals, laundering, going out for a short distance, using public transportation, shopping, managing money, making or taking a phone call, and taking medicine at the correct dosage and time [26]. The ADL/IADL scales were primary indicators for determining a participant’s functional disability [18]. In this study, participants were divided into persons without difficulties with ADL (or IADL) and those with at least one ADL (or IADL) limitation. The two-category variables were defined, with a value of “Yes” if the participant presented one or more limitations and a value of “No” if the participant did not show any limitations. This classification was consistent with the previous studies [7, 17].

### Demographic and health parameters

Demographic and health parameters at baseline (2008) were considered in our research criteria. Demographics included gender (male and female), age in years (65–69, 70–74, and 75 or older), living arrangement (with relatives and living alone), and educational status (elementary school or lower, middle school, high school, and college graduate or higher). Health parameters were body mass index (underweight, < 18.5 kg/m^2^; normal, 18.5–22.9 kg/m^2^; overweight, 23–25 kg/m^2^; and obese, > 25 kg/m^2^, based on the Asia-Pacific classification [28]), self-rated health (good and bad), difficulty in daily activities related to body pain (yes and no), physical activity (yes and no), depression status (yes and no, defined consistent with previous studies [29]), cognitive function, and the number of chronic diseases from the list of ten chronic diseases recorded: hypertension, diabetes mellitus, cancer, lung disease, liver disease, heart disease, cerebrovascular disease, psychiatric disorders, arthritis or rheumatism, and prostate disease. Cognitive function was determined through the Korean Mini-Mental State Examination (K-MMSE) [30]. This examination had a total of 30 possible scores; participants with higher scores showed better cognitive functioning. Cognitive function was classified into three levels, normal, cognitive decline, and dementia, corresponding to participants scoring more than 24, 18–24, and less than 18 on the K-MMSE, respectively [30, 31].

### Statistical analyses

We examined the change in the prevalence of ADL/IADL disability among older participants after every 4-year follow-up period from baseline (2008) through 2020 to verify the trend of this change. The change after 12 years was also demonstrated. Bivariate analyses were performed to identify participant characteristics and estimate the prevalence of ADL/IADL disability. Fisher’s exact test [32] calculated p-values for differences in ADL/IADL disability across participant characteristics. Differences in functional disability between males and females were also indicated by adjusting for age groups, cognitive function levels, living arrangements, and historical disability. To test the time-varying trend of changes in ADL/IADL disability, the generalized estimating equation (GEE) model [33] was performed. A p-value of less than 0.05 represents a statistically significant difference and an upward trend of ADL/IADL disability over time.

Multiple logistic regression models [34] were conducted to evaluate the influence of baseline characteristics on the presence of ADL/IADL disability, thereby demonstrating its change over time. In these models, the ADL/IADL disabilities during each 4-year follow-up period in 2012, 2016, and 2020 were considered to be the dependent variables, while predictors were demographics, health parameters, and functional disability at baseline. ADL and IADL disabilities at baseline were considered historical disabilities to predict future functional disability. The Wald test [34] was used to calculate the p-value for the parameter estimation and the likelihood-ratio Chi-square (LRC) test [35] assessed model fit. The “No” category of ADL/IADL disabilities was designated as the reference for the models. We reported the odds ratios (OR) obtained by exponentiation of the regression coefficients and the corresponding 95% confidence intervals (95% CI) for each model. The OR represented the relative risk ratio of ADL/IADL disability associated with a one-level change in the respective predictor. All analyses were conducted by using R software version 4.1.3.

## Results

### Baseline characteristics of participants

We included 1744 older adults, of which 1046 (approximately 60%) were female and 332 (19.04%) were oldest-old (aged 75 or older). The proportion of persons living alone was 26.55%. Those with elementary or lower education accounted for the highest percentage (approximately 68%), while college graduates or higher accounted for only 6.31%. Regarding health parameters, underweight persons accounted for a low rate (3.15%), while overweight or obese persons accounted for over 51%. Those with “bad” self-rated health or having difficulty in daily activities related to body pain accounted for significant rates (over 32%). Up to 63.47% of participants reported physical inactivity, 6.59% suffered from depression, approximately 35% experienced cognitive decline or dementia, and 68.23% had at least one chronic disease. The baseline characteristics of participants are shown in Table 1.

**Table 1:** Characteristics of participants at baseline. (2008)

Parameters at baseline (2008)	Number of participants (n)	Percentage (%)
**All participants**	1744	100.00
**Demographics**		
**Gender**		
Male	698	40.02
Female	1046	59.98
**Age in years**		
65–69	815	46.73
70–74	597	34.23
75 or older	332	19.04
**Living arrangement**		
With relatives	1281	73.45
Living alone	463	26.55
**Educational status**		
Elementary school or lower	1185	67.95
Middle school	201	11.53
High school	248	14.21
College or higher	110	6.31
**Health parameters**		
**Body mass index**		
Normal	797	45.70
Underweight	55	3.15
Overweight	503	28.84
Obese	389	22.31
**Self-rated health**		
Good	1147	65.77
Bad	597	34.23
**Difficulty in daily activities related to body pain**		
No	1177	67.49
Yes	567	32.51
**Physical activity**		
Yes	637	36.53
No	1107	63.47
**Depression status**		
No	1629	93.41
Yes	115	6.59
**Cognitive function**		
Normal	1139	65.31
Cognitive decline	445	25.52
Dementia	160	9.17
**Number of chronic diseases**		
0	554	31.77
1	618	35.43
2	377	21.62
≥ 3	195	11.18

*ADL* activities of daily living; *IADL* instrumental activities of daily living.

### Prevalence and trends of functional disability by baseline characteristics

The prevalence of ADL disability increased rapidly over time. The overall rate was 2.24% at baseline, increased to 3.10% after four years, 6.42% in 2016, and 11.12% in 2020. The change after 12 years was 8.88%. By gender, the upward trend over time occurred in both males and females, but there was no significant difference between males and females at baseline (p-value > 0.05). The prevalence of ADL disability in females increased more slowly than in males in 2012 but more rapidly in the following years. The change in prevalence of ADL disability after 12 years in males and females was 8.16% and 9.37%, respectively. By age group, the upward trend over time of ADL disability in persons aged 65–69 (1.47% at baseline, increased to 5.77% in 2020, 12-year change of 4.30%) was slower than in those aged 70–74 (2.18% at baseline, increased to 10.22% in 2020, 12-year change of 8.04%) and those aged 75 or older (4.22% at baseline, increased to 25.90% in 2020, 12-year change of 21.68%). By health parameters, the change in prevalence of ADL disability after 12 years was most significant among underweight and obese individuals (12.73% and 10.79%, respectively), while the upward trend over time of ADL disability presented more slowly in overweight individuals (2.19% at baseline, 6.16% increase after 12 years). The prevalence of ADL disability in those with “good” self-rated health was smaller and increased more slowly than in those with “bad” self-rated health (0.70% versus 5.19% at baseline, 7.67% versus 17.76% in 2020). Regarding cognitive function, the prevalence of ADL disability in persons with a normal level was the lowest, and the upward trend over time was also slower (1.14% at baseline, 6.50% increase after 12 years). In contrast, these rates in persons with dementia were substantially high and increased rapidly over time (7.50% at baseline, 29.38% in 2020). The prevalence of ADL disability increased rapidly in persons with at least three chronic diseases (4.62% at baseline, 16.92% in 2020). The prevalence of ADL disability and its trends are shown in Table 2.

**Table 2:** Prevalence of ADL disability and its trend over time among older adults in Korea

Parameters at baseline (2008)	Prevalence of ADL disability (%)				12-year change (%)	P-trend^†^
	2008	2012	2016	2020		
**All participants**	2.24	3.10	6.42	11.12	8.88	< 0.05
**Demographics**						
**Gender**						
Male	1.58	3.44	5.44	9.74	8.16	< 0.01
Female	2.68	2.87	7.07	12.05	9.37	< 0.05
P-diff^‡^	0.064	< 0.05	< 0.01	< 0.01		
**Age in years**						
65–69	1.47	1.84	4.02	5.77	4.30	< 0.05
70–79	2.18	2.35	4.05	10.22	8.04	< 0.05
75 or older	4.22	7.53	16.57	25.90	21.68	< 0.01
P-diff^‡^	< 0.05	< 0.001	< 0.001	< 0.001		
**Living arrangement**						
With relatives	1.41	2.58	5.31	9.13	7.72	< 0.05
Living alone	4.54	4.54	9.50	16.63	12.09	< 0.05
P-diff^‡^	< 0.001	< 0.05	< 0.01	< 0.001		
**Educational status**						
Elementary school or lower	2.62	3.38	7.34	12.83	10.21	< 0.05
Middle school	1.49	2.49	5.97	8.46	6.97	< 0.05
High school	1.21	2.82	3.63	6.85	5.64	< 0.05
College or higher	1.82	1.82	3.64	7.27	5.45	< 0.05
P-diff^‡^	0.231	0.375	< 0.05	< 0.01		
**Health parameters**						
**Body mass index**						
Normal	2.51	3.64	7.03	11.92	9.41	< 0.01
Underweight	0.01	1.82	7.27	12.73	12.72	< 0.01
Overweight	2.19	2.58	5.17	8.35	6.16	< 0.05
Obese	2.06	2.83	6.68	12.85	10.79	< 0.05
P-diff^‡^	0.331	0.328	0.259	< 0.05		
**Self-rated health**						
Good	0.70	1.48	3.92	7.67	6.97	< 0.05
Bad	5.19	6.20	11.22	17.76	12.57	< 0.05
P-diff^‡^	< 0.001	< 0.001	< 0.001	< 0.001		
**Difficulty in daily activities related to body pain**						
No	1.02	2.12	5.10	8.92	7.90	< 0.05
Yes	4.76	5.11	9.17	15.70	10.94	< 0.05
P-diff^‡^	< 0.001	< 0.001	< 0.001	< 0.001		
**Physical activity**						
Yes	1.10	2.51	5.81	9.58	8.48	< 0.05
No	2.89	3.43	6.78	12.01	9.12	< 0.05
P-diff^‡^	< 0.01	0.143	0.214	< 0.05		
**Depression status**						
No	1.96	3.07	6.26	10.74	8.78	< 0.05
Yes	6.09	3.48	8.70	16.52	10.43	0.113
P-diff^‡^	< 0.001	0.404	0.151	< 0.01		
**Cognitive function**						
Normal	1.14	1.76	4.48	7.64	6.50	< 0.05
Cognitive decline	3.15	3.82	7.19	13.48	10.33	< 0.05
Dementia	7.50	10.62	18.12	29.38	21.88	< 0.01
P-diff^‡^	< 0.001	< 0.001	< 0.001	< 0.001		
**Number of chronic diseases**						
0	0.72	1.26	3.25	7.04	6.32	< 0.05
1	2.27	2.91	7.77	11.81	9.54	< 0.05
2	3.18	4.77	6.90	13.00	9.82	< 0.05
≥ 3	4.62	5.64	10.26	16.92	12.30	< 0.05
P-diff^‡^	< 0.01	< 0.01	< 0.001	< 0.001		

*ADL* activities of daily living; † p-values for testing the upward trend of changes in ADL disability based on the generalized estimating equation model after controlling for demographics and health parameters; ‡ p-values for differences in ADL disability between specific categories using Fisher’s exact test

The prevalence of IADL disability and its trends are shown in Table 3. Unlike the ADL disability, the prevalence of IADL disability was much higher, tended to be flat during the first four years, and increased rapidly in the following years. The overall rate was 10.67% at baseline, 10.61% in 2012, 18.18% in 2016, and 25.57% in 2020. The change after 12 years was 14.90%. The prevalence of IADL disability was higher in males than in females during the first years (12.46% versus 9.46% at baseline, 12.75% versus 9.18% in 2012), but no significant differences were observed in subsequent years (around 18% in 2016 and over 25% in 2020). Differences were evident through age groups. The prevalence of IADL disability in persons aged 65–69 (6.75% at baseline, 14.36% in 2020) was significantly lower than in those aged 70–74 (10.72% at baseline; 28.31% in 2020), and 75 or older (20.18% at baseline, 48.19% in 2020). The prevalence of IADL disability in persons living alone was significantly higher than in those living with relatives in the period 2016–2020 (21.17% versus 17.10% in 2016, 30.67% versus 23.73% in 2020). However, there was almost no significant difference in the earlier years. The change after 12 years in persons living alone was 19.87% and in those living with relatives was 13.11%. The prevalence of IADL disability was lower in those with “good” self-rated health than in those with “bad” self-ratings (8.02% versus 15.75% at baseline, 20.58% versus 35.18% in 2020, respectively). There was a difference in the prevalence of IADL disability among levels of cognitive function. Persons with dementia had the highest rate of IADL disability (25.62% at baseline, 45% in 2020), while these rates in those with normal cognitive function were only 8.25% at baseline and 20.27% in 2020. The trend of IADL disability according to cognitive function or chronic diseases was consistent with the general trend being flat during the early years and then increasing rapidly.

**Table 3:** Prevalence of IADL disability and its trend over time among older adults in Korea

Parameters at baseline (2008)	Prevalence of IADL disability (%)				12-year change (%)	P-trend^†^
	2008	2012	2016	2020		
**All participants**	10.67	10.61	18.18	25.57	14.90	< 0.05
**Demographics**						
**Gender**						
Male	12.46	12.75	18.05	25.07	12.61	< 0.05
Female	9.46	9.18	18.26	25.91	16.45	< 0.05
P-diff^‡^	< 0.05	< 0.01	0.452	0.324		
**Age in years**						
65–79	6.75	6.26	11.66	14.36	7.61	0.062
60–74	10.72	10.72	17.42	28.31	17.59	< 0.05
75 or older	20.18	21.08	35.54	48.19	28.01	< 0.05
P-diff^‡^	< 0.001	< 0.001	< 0.001	< 0.001		
**Living arrangement**						
With relatives	10.62	10.54	17.10	23.73	13.11	0.052
Living alone	10.80	10.80	21.17	30.67	19.87	< 0.05
P-diff^‡^	0.813	0.867	< 0.05	< 0.01		
**Educational status**						
Elementary school or lower	10.97	10.46	19.41	28.02	17.05	0.061
Middle school	11.44	10.45	16.42	19.90	8.46	0.073
High school	8.87	10.89	15.32	20.16	11.29	< 0.01
College or higher	10.00	11.82	14.55	21.82	11.82	< 0.05
P-diff^‡^	0.384	0.487	0.133	< 0.01		
**Health parameters**						
**Body mass index**						
Normal	5.45	5.45	23.64	27.27	21.82	< 0.05
Underweight	9.91	10.41	18.82	27.10	17.19	< 0.01
Overweight	11.53	10.74	15.11	20.68	9.15	0.053
Obese	11.83	11.57	20.05	28.53	16.70	< 0.05
P-diff^‡^	0.193	0.291	< 0.05	< 0.01		
**Self-rated health**						
Good	8.02	7.76	13.60	20.58	12.56	0.061
Bad	15.75	16.08	26.97	35.18	19.43	< 0.05
P-diff^‡^	< 0.001	< 0.001	< 0.001	< 0.001		
**Difficulty in daily activities related to body pain**						
No	8.24	8.92	15.46	22.68	14.44	< 0.05
Yes	15.70	14.11	23.81	31.57	15.87	< 0.05
P-diff^‡^	< 0.001	< 0.001	< 0.001	< 0.001		
**Physical activity**						
Yes	9.26	9.11	17.58	23.55	14.29	< 0.05
No	11.47	11.47	18.52	26.74	15.27	< 0.05
P-diff^‡^	< 0.05	< 0.05	0.213	< 0.05		
**Depression status**						
No	10.50	10.62	17.99	25.48	14.98	< 0.05
Yes	13.04	10.43	20.87	26.96	13.92	0.063
P-diff^‡^	0.146	0.475	0.069	0.262		
**Cognitive function**						
Normal	8.25	8.25	14.14	20.72	12.47	< 0.05
Cognitive decline	11.46	11.24	21.80	31.01	19.55	0.051
Dementia	25.62	25.62	36.88	45.00	19.38	< 0.05
P-diff^‡^	< 0.001	< 0.001	< 0.001	< 0.001		
**Number of chronic diseases**						
0	7.22	7.22	14.44	22.92	15.70	0.051
1	11.00	10.36	16.18	25.89	14.89	0.051
2	11.94	13.53	20.95	25.73	13.79	< 0.05
≥ 3	16.92	15.38	29.74	31.79	14.87	0.052
P-diff^‡^	< 0.001	< 0.001	< 0.001	< 0.01		

*IADL* instrumental activities of daily living; † p-values for testing the upward trend of changes in IADL disability based on the generalized estimating equation model after controlling for demographics and health parameters; ‡ p-values for differences in IADL disability between specific categories using Fisher’s exact test

**Fig. 2:**
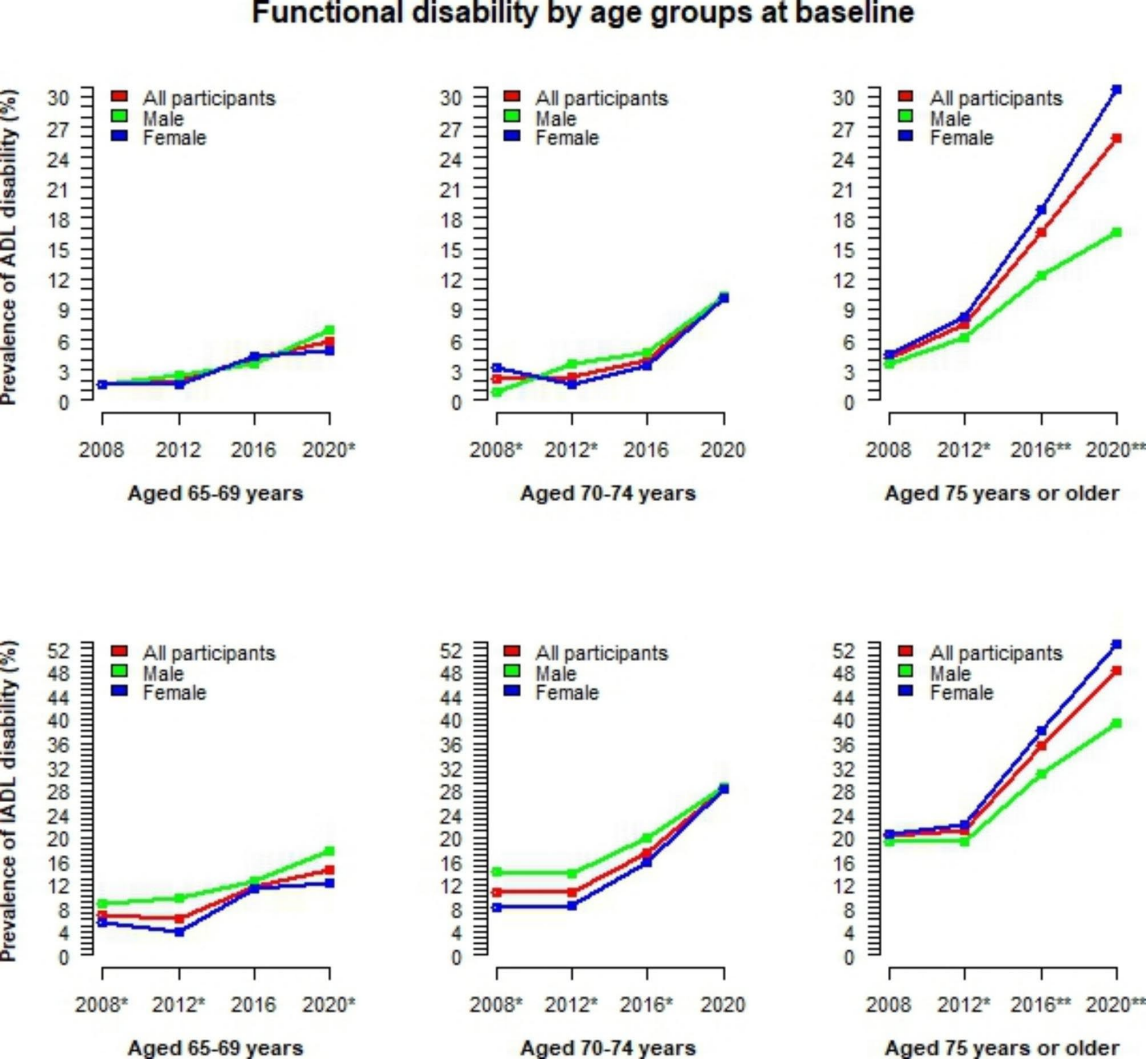
Time-varying trends of functional disability by gender and age groups *ADL* activities of daily living; *IADL* Instrumental activities of daily living; Fisher’s exact test was used to calculate p-values for differences between males and females; ‘***’ p<0.001, ‘**’ p<0.01, ‘*‘ p<0.05, ‘ ’ p≥0.05

**Fig. 3:**
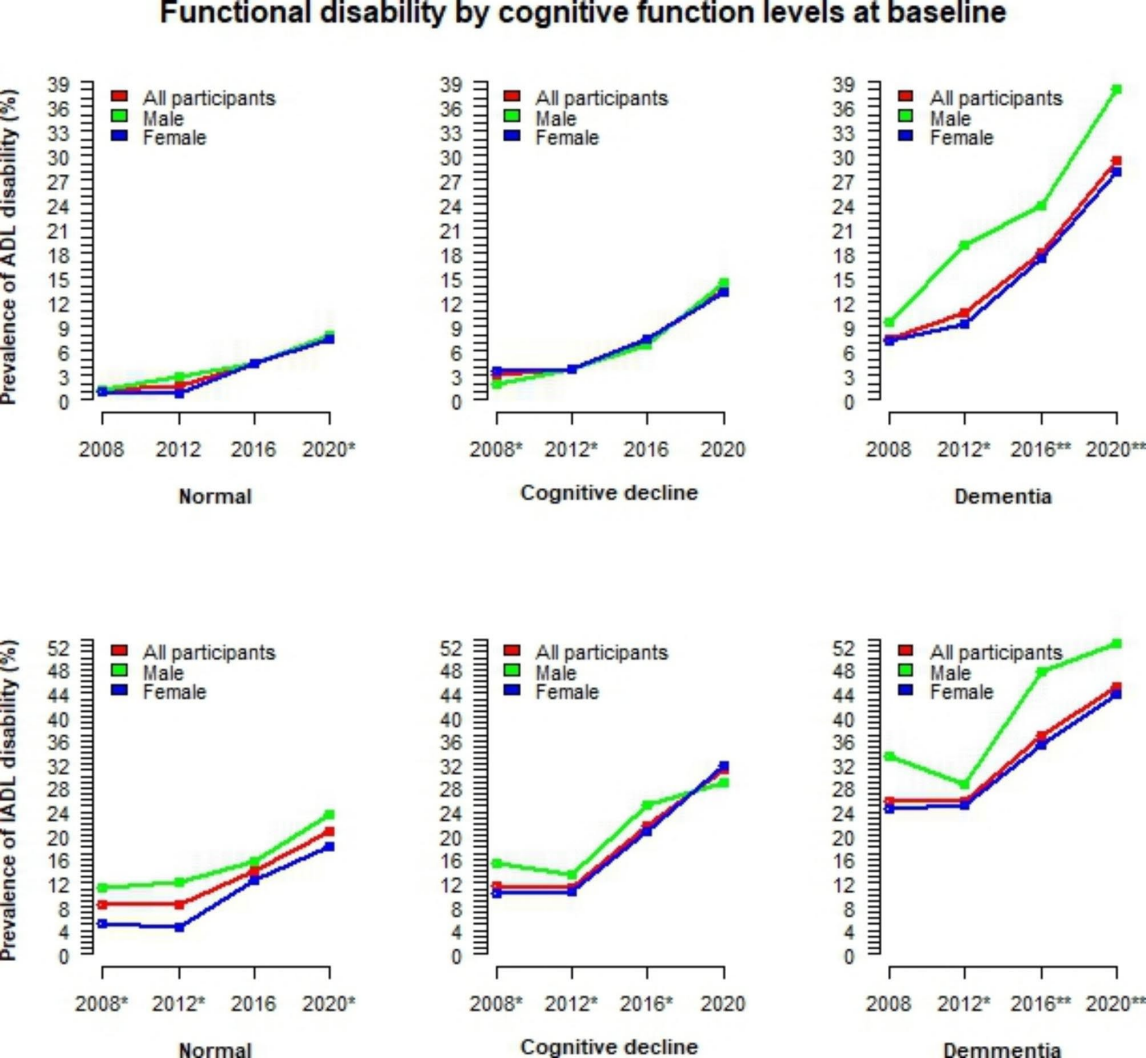
Time-varying trends of functional disability by gender and cognitive function levels *ADL* activities of daily living; *IADL* Instrumental activities of daily living; Fisher’s exact test was used to calculate p-values for differences between males and females; ‘***’ p<0.001, ‘**’ p<0.01, ‘*’ p<0.05, ‘ ’ p≥0.05

**Fig. 4:**
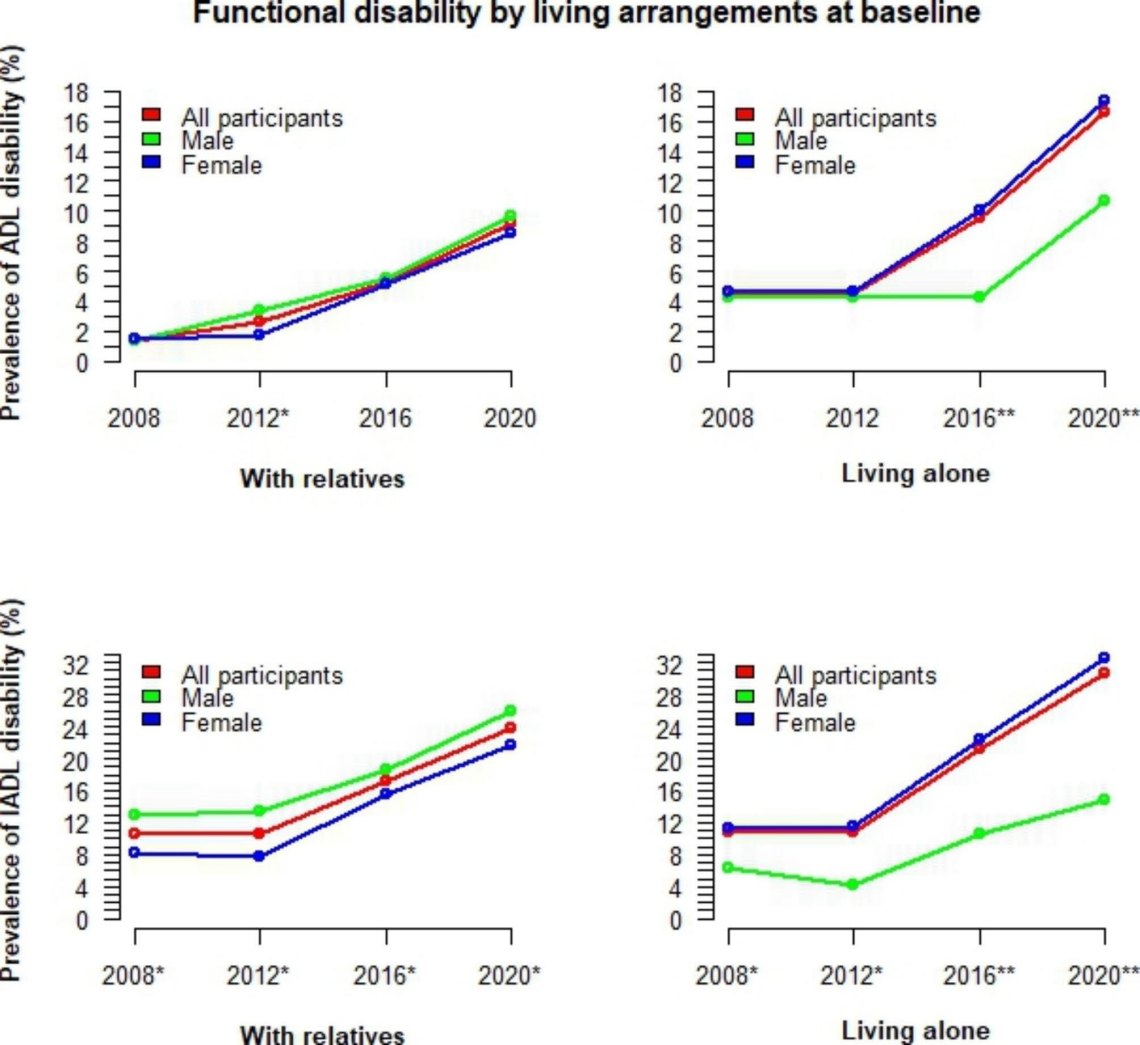
Time-varying trends of functional disability by gender and living arrangements *ADL* activities of daily living; *IADL* Instrumental activities of daily living; Fisher’s exact test was used to calculate p-values for differences between males and females; ‘***’ p<0.001, ‘**’ p<0.01, ‘*’ p<0.05, ‘ ’ p≥0.05

The time-varying trends of functional disability by gender and age groups are illustrated in Fig. 2. For those under 75 years old, ADL disability increased slowly over time, and there was no significant difference between males and females. In contrast, for persons aged 75 or older, ADL disability increased rapidly. For IADL disability, a flat trend in the earlier years was evident in all age groups, followed by a slow increase in persons aged 65–69 and a rapid increase in those aged 70 or older. In particular, for persons aged 75 or older, the ADL/IADL disability rate in males was consistently lower and increased more slowly than in females. In contrast, for persons under 75 years old, the rate of IADL disability in males was higher. The changes in functional disability among older adults by cognitive function levels and living arrangements are also visualised in Figs. 3 and 4. Among those with dementia, the rate of functional disability is significantly higher in males than in females. In contrast, this rate is significantly lower in males living alone than in females living alone.

**Fig. 5:**
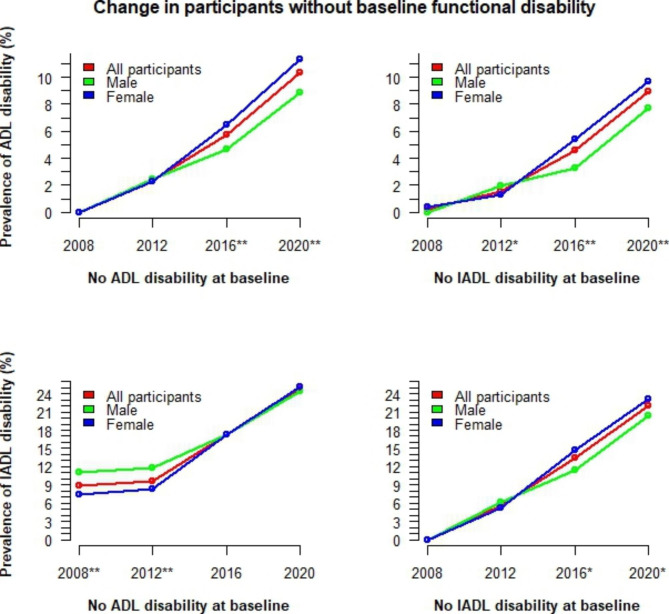
Changes over time of functional disability in participants without baseline functional disability by gender *ADL* activities of daily living; *IADL* Instrumental activities of daily living; Fisher’s exact test was used to calculate p-values for differences between males and females; ‘***’ p<0.001, ‘**’ p<0.01, ‘*’ p<0.05, ‘ ’ p≥0.05

The change over time of ADL/IADL disability in persons without disabilities at baseline is illustrated in Fig. 5. The prevalence of ADL disability increased rapidly in both males and females, and no significant difference was observed between persons without ADL disability or IADL disability at baseline. This was distinct from the IADL disability. For those without ADL disability at baseline, the rate of IADL disability at baseline was still significant. This rate tended to be flat during the early years and then increased rapidly, while for those without IADL disability at baseline, IADL disability increased consistently.

#### Multiple logistic regression analyses

The results of multiple logistic regression analyses are shown in Table 4. The 5% significance level represented the statistically significant attributes in the models. The small p-values from LRC tests (p-values < 0.00001) demonstrated the adequacy and robustness of the models.

**Table 4:** Results of multiple logistic regression analyses for ADL/IADL disability

Parameters at baseline (2008)	ADL disability			IADL disability		
	2012 OR^†^ (95% CI)	2016 OR^†^ (95% CI)	2020 OR^†^ (95% CI)	2012 OR^†^ (95% CI)	2016 OR^†^ (95% CI)	2020 OR^†^ (95% CI)
Demographics						
**Gender** (Reference category: Male)						
Female	0.685** (0.416;0.867)	1.278* (1.032;1.549)	1.378* (1.039;1.746)	0.537** (0.481;0.623)	1.108 (0.821;1.454)	1.215 (0.867;1.568)
**Age in years** (Reference category: 65–69)						
70–74	1.335* (1.098;1.622)	1.343* (1.172;1.538)	1.486* (1.245;1.642)	1.281* (1.104;1.485)	1.426* (1.283;1.683)	2.087** (1.753;2.331)
75 or older	1.882*** (1.556;2.275)	2.924*** (2.584;3.308)	3.677*** (3.223;4.069)	2.619*** (2.332;2.937)	2.925*** (2.586;3.386)	4.381*** (3.861;4.927)
**Living arrangement** (Reference category: With relatives)						
Living alone	1.623** (1.356;1.943)	1.352* (1.203;1.524)	1.528** (1.395;1.674)	1.012 (0.903;1.129)	1.072 (0.989;1.161)	1.182 (0.904;1.266)
**Educational status** (Reference category: College or higher)						
Elementary school or lower	1.883*** (1.243;2.853)	1.013 (0.749;1.374)	1.099 (0.883;1.367)	1.698*** (1.411;2.044)	1.026 (0.878;1.199)	1.017 (0.894;1.157)
Middle school	1.548** (1.103;2.087)	1.696*** (1.264;2.275)	1.085 (0.871;1.353)	1.324* (1.078;1.627)	1.106 (0.931;1.313)	1.214 (0.949;1.406)
High school	1.202* (1.071;1.493)	1.295* (1.086;1.502)	1.133 (0.929;1.382)	1.101 (0.906;1.337)	1.144 (0.969;1.353)	1.088 (0.947;1.251)
**Health parameters**						
**Body mass index** (Reference category: Normal)						
Underweight	1.652** (1.102;2.615)	1.075 (0.822;1.407)	1.032 (0.838;1.271)	1.975*** (1.442;2.705)	1.451* (1.224;1.716)	1.058 (0.904;1.238)
Overweight	1.331* (1.113;1.589)	1.321* (1.167;1.495)	1.427* (1.294;1.574)	1.067 (0.963;1.181)	1.342* (1.238;1.455)	1.379* (1.287;1.477)
Obese	1.814** (1.492;2.209)	1.292* (1.133;1.469)	1.024 (0.927;1.133)	1.011 (0.902;1.134)	1.071 (0.983;1.166)	1.075 (0.903;1.256)
**Self-rated health** (Reference category: Good)						
Bad	2.654*** (2.225;3.166)	2.437*** (2.164;2.744)	1.834*** (1.671;2.013)	1.903*** (1.713;2.115)	1.904*** (1.759;2.062)	1.892*** (1.764;2.025)
**Difficulty in daily activities related to body pain** (Reference category: No)						
Yes	1.405* (1.127;1.816)	1.228* (1.088;1.385)	1.064 (0.968;1.171)	1.312* (1.106;1.626)	1.051 (0.968;1.142)	1.008 (0.941;1.082)
**Physical activity** (Reference category: Yes)						
No	2.169*** (1.692;3.381)	1.628** (1.399;2.171)	1.471* (1.182;1.869)	1.757** (1.251;2.275)	1.336* (1.152;1.528)	1.159* (1.095;1.326)
**Depression status** (Reference category: No)						
Yes	2.295*** (1.713;3.084)	2.132*** (1.443;2.861)	2.081*** (1.339;2.744)	1.659** (1.381;1.993)	1.317** (1.155;1.501)	1.323** (1.176;1.482)
**Cognitive function** (Reference category: Normal)						
Cognitive decline	1.687** (1.396;2.043)	1.441* (1.118;1.484)	1.362* (1.238;1.498)	1.301* (1.165;1.452)	1.454* (1.342;1.575)	1.397* (1.306;1.495)
Dementia	3.211*** (2.583;3.992)	1.914*** (1.652;2.217)	2.561*** (2.277;2.882)	2.492*** (2.169;2.863)	2.088*** (1.873;2.328)	1.834*** (1.664;2.022)
**Number of chronic diseases** (Reference category: 0)						
1	1.593** (1.269;1.999)	1.952** (1.694;2.253)	1.423* (1.281;1.581)	1.165* (1.039;1.307)	1.147* (1.053;1.249)	1.068 (0.996;1.146)
2	2.366*** (1.864;3.004)	1.369* (1.161;1.615)	1.337* (1.185;1.507)	1.503** (1.321;1.709)	1.122* (1.017;1.233)	1.263* (1.162;1.374)
≥ 3	2.491*** (1.912;3.254)	1.862** (1.555;2.231)	1.619** (1.411;1.858)	1.895*** (1.195;2.527)	1.611** (1.441;1.803)	1.462* (1.159;1.675)
**Functional disability at baseline**						
**ADL disability at baseline** (Reference category: No)						
Yes	4.155*** (3.331;5.182)	2.271*** (1.857;2.775)	2.134*** (1.767;2.578)	2.061*** (1.379;2.882)	1.668** (1.369;2.107)	1.261* (1.082;1.476)
**IADL disability at baseline** (Reference category: No)						
Yes	4.709*** (3.976;5.577)	3.118*** (2.751;3.534)	2.291*** (2.057;2.551)	13.675*** (12.410;15.071)	7.094*** (6.474;7.772)	3.091*** (2.829;3.376)

*ADL* activities of daily living; *IADL* instrumental activities of daily living; † The odds ratio (OR) and the corresponding 95% confidence intervals (95% CI) were calculated against no ADL/IADL disability as the reference; ‘***’ p < 0.001, ‘**’ p < 0.01, ‘*’ p < 0.05, ‘ ’ p ≥ 0.05

The obtained results showed the differential influence of parameters at baseline on ADL/IADL disability among 4-year follow-up periods in 2012, 2016, and 2020. ADL disability in 2012 was significantly associated with all demographic and health parameters at baseline (p-values < 0.05). The factors that had strong influence on the change of ADL disability in all 4-year follow-up periods (all p-values < 0.05) were age of 75 years or older (OR = 3.381, 95% CI = 3.042–3.758, p-value < 0.001 in 2016, living alone (OR = 1.623, 95% CI = 1.356–1.943, p-value < 0.01 in 2012), poor health status (OR = 2.654, 95% CI = 2.225–3.166, p-value < 0.001 in 2012), physical inactivity (OR = 2.169, 95% CI 1.692–3.381, p-value < 0.001 in 2012), depression (OR = 2.295, 95% CI = 1.713–3.084, p-value < 0.001 in 2012, dementia (OR = 3.211, 95% CI = 2.583–2.992, p-value < 0.001 in 2012), three or more chronic diseases (OR = 2.491, 95% CI = 1.912–3.254, p-value < 0.001 in 2012), and historical ADL disability (OR = 4.155, 95% CI = 3.331–5.182, p-value < 0.001 in 2012). Historical ADL disability was the strongest factor among all parameters. Similar to ADL disability, IADL disability in 2012 was significantly associated with most demographic and health parameters at baseline, except for living alone, having high school education, and being overweight or obese. The factors that strongly predicted the change of IADL disability in all 4-year follow-up periods were age of 75 or older (OR = 2.968, 95% CI = 2.779–3.169, p-value < 0.001 in 2020, poor health status (OR = 1.904, 95% CI = 1.759–2.062, p-value < 0.001 in 2016, physical inactivity (OR = 1.757, 95% CI = 1.251–2.275, p-value < 0.01 in 2012), depression (OR = 1.659, 95% CI = 1.381–1.993, p-value < 0.01 in 2012), dementia (OR = 2.492, 95% CI = 2.169–2.863, p-value < 0.001 in 2012), three or more chronic diseases (OR = 1.895, 95% CI = 1.195–2.527, p-value < 0.001 in 2012), and historical IADL disability (OR = 13.675, 95% CI = 12.41–15.07, p-value < 0.001 in 2012).

## Discussion

Functional disability in older adults has presented many obstacles to policymakers and national healthcare systems, especially in recent years, as global population aging has taken place rapidly [4, 5]. Therefore, determining the prevalence of functional disability and its trend over time in older adults was essential [8]. In this study, we performed a 4-year follow-up cohort study to show the trend over time of functional disability and to determine factors affecting the change in prevalence of functional disability. It has been relatively new and scarce, especially for older Korean adults. Our findings were important for formulating social policies and improving the Korean healthcare system to properly accommodate the development of functional disabilities among older adults. This study used data from the Korean Longitudinal Study of Aging (KLOSA) surveys.

Our study found that prevalence of ADL disability increased slowly in the earlier years while IADL disability was stable. The prevalence of these disabilities then tended to increase rapidly over time, especially in persons aged 75 or older. The upward trends for both ADL and IADL disability were statistically significant. The results on disability trends were consistent with previous studies [6, 8, 25]. One of the strengths of this study was to show that disability trends progressed in older adults over a long period. In contrast, previous studies based on a short follow-up only showed significant trends in the early stages of disability development. In such a different scenario, the GEE model [33, 36] was performed to obtain an improved significance test. Our finding has suggested for policymakers targeting older adults, especially those with functional disabilities; a long-term strategy is needed to monitor and care for them from the early years, thereby minimizing the significant increase in functional disability as they age.

The ADL and IADL scales both identified functional disability but measured different aspects, so trends for IADL disability differed from trends for ADL disability [6]. Therefore, studying the trend of these disabilities separately was necessary. As shown in this study, for persons without ADL disability at baseline, gender differences in ADL and IADL disabilities showed that there was no significant difference in ADL disability, but significant in IADL disability at baseline. These trends were reversed in both eight years and 12 years later. For persons without IADL disability, gender was not significantly different in both ADL and IADL disability at baseline, but these trends failed to be maintained in later years. Because of a new trial in examining the trends of gender differences in ADL and IADL disabilities according to no ADL and IADL disabilities at baseline, interpreting these findings using the existing studies was difficult. Despite the interpretation limitation, future research is needed to support the findings.

This study found factors strongly associated with the change of functional disability during a long follow-up period. These factors included advanced age, living alone, poor health status, physical inactivity, depression, dementia, multiple chronic diseases, and a history of disability. Especially significant 12-year changes in ADL disability were observed in persons aged 75 or older (21.68%), persons living alone (12.09%), those with poor health status (12.57%), and persons with dementia (21.88%). Similar to IADL disability, the change was 28.01% for those aged 75 or older, 19.87% for persons living alone, 19.43% for those with poor health status, and 19.38% for persons with dementia. Except for historical disability, these factors were consistent with and were reported in previous studies related to functional disability [7–9, 11, 13, 15–17]. Physical inactivity led to psychosis and dependence when performing activities [38]. Depressive states and dementia increased the risk of further disability [7, 16], while persons with two or more chronic diseases faced an early functional decline [17]. Modification of physical activity, participation in social activities, the help of caregivers, and an early diagnosis of chronic conditions were essential to the establishment of care and rehabilitation plans for older adults.

Some interesting findings in this study were related to living arrangements and educational status of those with IADL disability changes. There was no significant difference in IADL disability either between living with relatives and living alone or among educational levels at baseline; however, both living alone and elementary or lower education groups significantly changed in IADL disability after 12 years. Although social-economic parameters including educational status in older adults have been well known as the strong factors for health, living alone and elementary or lower education groups demonstrated significant changes in IADL disability. The prevalence of older adults living alone is currently globally increasing, especially for older male adults [37]. These findings have suggested future analysis of the changes in functional disability in this area of phenomena.

We found that the prevalence of functional disability in the underweight group was higher and tended to increase more rapidly than in the overweight group, whereas it was lower and increased more slowly than in the obese group. This finding is different and has not been explained by previous studies from Korea that BMI had a negligible effect on ADL/IADL disability among Korean older adults [39, 40]. A recent interesting study has shown a u-shaped relationship between functional disability and BMI in older adults [41]. Accordingly, both underweight and obesity, including abdominal obesity, were high-risk factors for increased ADL/IADL disability, while being overweight tends to correlate with decreased ADL disability. This result once again confirmed the robustness of our findings.

Our findings also indicated that the overall prevalence of ADL disability was lower in males than in females. However, among those with dementia, males tended to have higher ADL and IADL problems. This has also been presented in previous studies [42]. Although the prevalence of dementia in males was much lower than in females [43], it has not been clear why they tended to be more dependent than females. A further study of functional disability in those with gender-related dementia should be performed in the future.

In this study, historical disability was considered an essential factor affecting the progress of future functional disability. This aspect has not been studied comprehensively and systematically in the previous studies. Our study found that historical disability was the strongest factor among parameters that predicted the change in later disability. Specifically, the relative risk of ADL disability development in persons with an ADL disability at baseline was increased more than four times after four years compared with those without ADL disability at baseline. The number in those with IADL disability at baseline was 4.7 times higher than those without IADL disability at baseline. The same was true for IADL disability. This showed that early identification of historical disability was influential in reducing the development of severe disability.

Our study had several limitations. First, this study did not include several parameters such as living region, income, smoking status, and alcohol consumption to minimize missing data. Second, this study only focused on older adults who participated in all surveys over a long period of time, so it omitted an important group including those who lost follow-up due to death or discontinuation of the process. However, it is difficult to further include this group in the analysis due to the lack of relevant information. In addition to the unreported deaths, a few persons, despite their good health, for personal reasons did not continue to participate. Third, to reduce complexity, this study was only concerned with the presentation of functional disability in general, not with regard to the distribution of the number of ADL and IADL items. Lastly, instead of assessing the influence of each chronic disease on functional disability, we only looked at the existing number of chronic diseases. This was convenient for the presentation of research results. A comprehensive and systematic study of the differential influence of chronic diseases on functional disability in general and each item of functional disability in particular is necessary for future studies.

## Conclusion

The study verified the change in functional disability and its uptrend over time by older adults’ demographic and health parameters. Functional disability tended to increase slowly, or flatly, during the earlier years but increased rapidly in the following years. Factors strongly influencing the change in prevalence and the upward trend of functional disability over time were advanced age, living alone, being underweight or obese, poor health status, physical inactivity, depression, dementia, multiple chronic diseases, and especially a history of functional disability.

## Data Availability

The datasets analysed during the current study are available in the repository [26], [https://survey.keis.or.kr/eng/klosa/klosa01.jsp]

## List of Abbreviations

ADL: Activities of daily living
CI: Confidence interval
IADL: Instrumental activities of daily living
GEE: Generalized estimating equation
K-MMSE: Korean Mini-Mental State Examination
KLOSA: Korean Longitudinal Study of Aging
LRC: Likelihood-ratio Chi-square test
OR: Odds ratio
